# The BOADICEA model of genetic susceptibility to breast and ovarian cancer

**DOI:** 10.1038/sj.bjc.6602175

**Published:** 2004-09-21

**Authors:** A C Antoniou, P P D Pharoah, P Smith, D F Easton

**Affiliations:** 1 Cancer Research UK, Genetic Epidemiology Unit, Strangeways Research Laboratory, Department of Public Health and Primary Care, Worts Causeway, Cambridge CB1 8RN, UK; 2 Cancer Research UK, Human Cancer Genetics Group, Department of Oncology, University of Cambridge, Cambridge, UK

**Keywords:** genetic counselling, BRCA1/2, risk modifiers, polygenic model

## Abstract

Several genes conferring susceptibility to breast and ovarian cancer, notably BRCA1 and BRCA2, have been identified. The majority of the familial aggregation of breast cancer is, however, not explained by these genes. We have previously derived, using segregation analysis, a susceptibility model (BOADICEA, Breast and Ovarian Analysis of Disease Incidence and Carrier Estimation Algorithm) in which susceptibility to these genes is explained by mutations in BRCA1 and BRCA2 together with a polygenic component reflecting the joint multiplicative effect of multiple genes of small effect on breast cancer risk. Here, we consider the predictions made by this model. The overall familial risks of breast cancer predicted by this model are close to those observed in epidemiological studies. The predicted prevalences of BRCA1 and BRCA2 mutations among unselected cases of breast and ovarian cancer are also consistent with observations from population-based studies. These predictions are closer to the observed values than those obtained using the Claus model and BRCAPRO. The predicted mutation probabilities and cancer risks in individuals with a family history (FH) can differ markedly from those predicted by other models. We conclude that this model provides a rational basis for risk assessment in individuals with a FH of breast or ovarian cancer.

Genetic testing for the breast and ovarian cancer susceptibility genes BRCA1 and BRCA2 can help in the clinical management of individuals with family history (FH) of the disease, by identifying individuals at highest risk. Those individuals can then be offered screening from an earlier age, prophylactic mastectomy or oophorectomy or potentially chemoprevention, for example, with tamoxifen (Hartmann *et al*, 2001; Meijers-Heijboer *et al*, 2001; King *et al*, 2001). However, genetic testing is expensive and may be associated with adverse psychosocial effects (Burke *et al*, 1997; Davis, 1997). Therefore, to provide an effective genetic counselling service, it is important that genetic testing is targeted towards those individuals most likely to prove positive.

Mathematical models, which predict carrier probabilities and cancer risks, can provide a rational basis for counselling. A number of models, which address the problem of predicting the BRCA1 and BRCA2 carrier risks and/or the breast and ovarian risks have been reported in the literature (Gail *et al*, 1989; Claus *et al*, 1991, 1994; Shattuck-Eidens *et al*, 1995; Couch *et al*, 1997; Parmigiani *et al*, 1998; Apicella *et al*, 2003). Several of these are logistic regression models that utilise descriptive measures of FH. Such models are straightforward to implement and have the advantage that other nongenetic risk factors can readily be incorporated (Gail *et al*, 1989; Shattuck-Eidens *et al*, 1995; Couch *et al*, 1997; Apicella *et al*, 2003). The disadvantage with this approach is that it cannot deal adequately with complex family histories. An alternative approach is to base predictions on a genetic model for the disease. The first genetic model to be widely used was developed by Claus *et al* (1991, 1994). This allows for a single highly penetrant gene, which is dominantly inherited. A more recent model, developed by Parmigiani *et al* (1998) and implemented in the computer software BRCAPRO (Berry *et al*, 2002) allows for the simultaneous effect of BRCA1 and BRCA2. However, recent research has indicated that genetic susceptibility to breast cancer is more complex than these models suggest. Epidemiological studies have demonstrated that BRCA1 and BRCA2 mutations account for less than 20% of the familial aggregation of breast cancer (Easton 1999; Peto *et al*, 1999). An adequate genetic model for breast cancer must clearly reflect the existence of other susceptibility genes.

In a previous article, we described the development of a genetic model for familial breast cancer, which takes into account the simultaneous effects of BRCA1, BRCA2 and other genes (Antoniou *et al*, 2002). In this article, we investigate the predictions of this model. We compute cancer risks and mutation carrier probabilities for a variety of different scenarios. The predicted results are compared with those from epidemiological studies and contrasted with the results of other models.

## MATERIALS AND METHODS

### The model

The analyses in this article are based on the best fitting model found by Antoniou *et al* (2002). This model was developed using complex segregation analysis of breast and ovarian cancer occurrence in a combined data set of a population-based series of 1484 breast cancer cases and 156 multiple-case families. Briefly, the model allows for the simultaneous effects of BRCA1, BRCA2, with disease allele frequencies 0.051% and 0.065%, respectively, and the effect of low-penetrance genes with multiplicative effects on the breast cancer risk (Antoniou *et al*, 2002). It also allows for the effect of genetic modifiers, which cluster in families and alter the breast cancer risks in BRCA1 and BRCA2 carriers. Under this model, the incidence of breast cancer at age *t* is given by


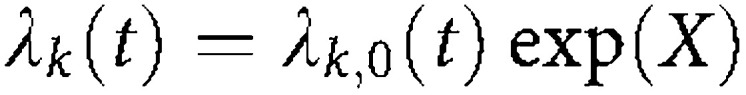


where *k*=0,1,2 for noncarriers, BRCA1 and BRCA2 carriers, respectively. *X* is a polygenic component, assumed to be normally distributed with mean 0 and variance *σ*^2^ and is independent of age. *σ*^2^ is estimated to be 1.67; it is assumed to be the same in BRCA1 and BRCA2 carriers and noncarriers (Antoniou *et al* (2002) found no significant difference in *σ*^2^ between carriers and noncarriers). Ovarian cancer rates follow a similar model but with no polygenic component. Other cancers are ignored in this model. The baseline incidence rates λ_*k*,0_(*t*) are given in Table 1

**Table 1:** Baseline BRCA1, BRCA2 and noncarriers' breast cancer incidence rates and corresponding ovarian cancer incidence rates used in the proportional-hazards model (Antoniou *et al*, 2002)

	**Baseline breast cancer incidence rate (%)**			**Ovarian cancer incidence rate (%)**		
**Age (years)**	**Noncarriers**	**BRCA1**	**BRCA2**	**Noncarriers**	**BRCA1**	**BRCA2**
30	0.009	0.538	0.375	0.004	0.021	0.022
40	0.040	1.021	0.799	0.012	1.173	0.044
50	0.068	0.677	1.484	0.031	0.813	0.462
60	0.092	0.450	2.612	0.044	0.976	0.416
70	0.114	0.481	3.591	0.048	0.100	0.100

. These incidence rates are chosen so that the overall age-specific incidence for both diseases, averaged over all genotypes, is constrained to agree with population incidence rates – in this implementation these are the rates for England and Wales over the period 1983–1987. Ovarian and breast cancer risks are assumed to be independent given the genotype. In the current implementation, only females are susceptible to breast cancer. The cumulative breast cancer risk, averaged over all possible modifiers, is 35% by age 70 years for BRCA1 carriers and 50% for BRCA2 carriers; the corresponding ovarian cancer risks are 26% for BRCA1 carriers and 9.1% for BRCA2 carriers. Nonmutation carriers have a cumulative risk of 5% of developing breast cancer and 1% probability of developing ovarian cancer by age 70 years. The model is implemented in the pedigree analysis computer program MENDEL v3.3 (Lange *et al*, 1988). This program allows genotype probabilities and cancer risks to be derived for pedigrees of arbitrary structure. We have named this model BOADICEA (Breast and Ovarian Analysis of Disease Incidence and Carrier Estimation Algorithm). The routines to implement this model are available from the authors on request.

Information on testing for BRCA1 and BRCA2 mutations can also be taken into account. In this implementation, mutations are assumed to be clearly pathogenic (i.e. mutation testing is 100% specific) but mutation detection sensitivity can be varied. In the examples presented here, the sensitivity is assumed to be 100% (that is, the probabilities refer to the probabilities of a mutation being present, not necessarily the probabilities of a mutation being detected). The possibility of both BRCA1 and BRCA2 mutations in the same individual is ignored.

### Carrier probabilities

The probability that an individual carries a BRCA1 or a BRCA2 mutation based on the FH information can be computed using Bayes theorem. For example,


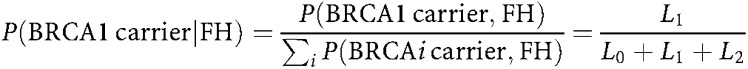


where *L*_*i*_ is the likelihood of observing the family with the index individual carrying mutation *i* (=0, 1, 2 for mutation negative, BRCA1 and BRCA2, respectively). These likelihoods can be generated in MENDEL.

### Cancer risks

We computed the probability that a woman of a given age *x* years developed breast or ovarian cancer by age *x*+*n*, given the known FH. For example, if we denote the event of breast cancer by BC and the event of being unaffected by *U*, then


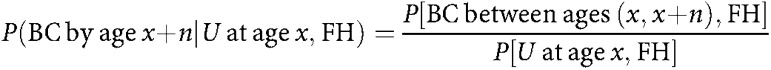


The denominator corresponds to the likelihood of observing the pedigree with the individual being unaffected at age *x*. The numerator is equivalent to the likelihood of observing the family, but replacing the penetrance for the index case by


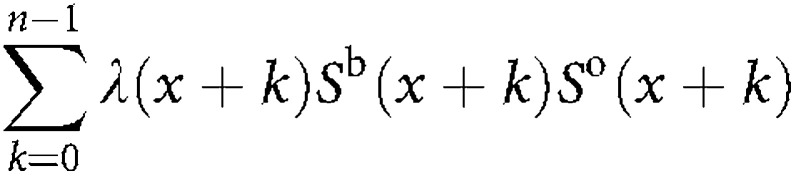


where λ*(x)* is the breast cancer incidence at age *x*, and *S*^b^(t) and *S*^o^(t) are the probabilities of remaining free of breast and ovarian cancer respectively by age *t.* Hence, the risks can be computed as the ratio of two likelihoods.

### Breast cancer familial relative risks (FRRs)

We define the FRR of breast cancer to a relative of a breast cancer patient to be the ratio of the risk to the relative to the population risk (Risch, 1990). The FRR predicted by the model can also be computed from two likelihoods using MENDEL. For example, in the case of FRR to daughters


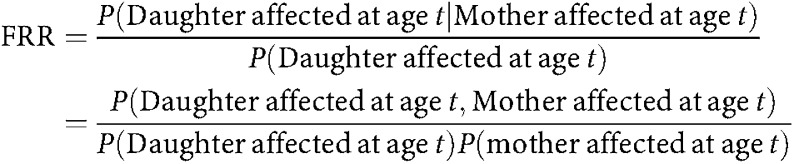


Note that all probabilities refer to the density of the risk distribution (i.e. probability of being affected at age *t*) rather than the incidence rates. These probabilities are therefore comparable to those derived from case–control studies. The FRR calculated above varies by age. To calculate an overall FRR, the age-specific FRRs were averaged over ages 20–70 years, weighted by the age distribution of breast cancer cases in the population.

### Risk of contralateral breast cancer

The segregation analysis used to derive the BOADICEA model considered only the occurrence of the first cancer in an individual (breast or ovary), and does not therefore make any direct predictions about the risks of a contralateral breast cancer. Predictions can be made, however, if one assumes that the increased risks of contralateral breast cancer (relative to population rates) are entirely due to susceptibility as defined by the model, that is, there is no additional individual variation in risk. Under this model, the contralateral breast cancer incidence rate after the first breast cancer, given the genotype, is half the incidence rate assumed in the standard model, to allow for only one breast being at risk. Ovarian cancer incidence rates after a breast cancer are assumed to be the same as if the breast cancer had not occurred (conditional on genotype) consistent with the assumption of independence between breast and ovarian cancer risks.

## RESULTS

### Mutation carrier probabilities

Table 2

**Table 2:** Predicted BRCA1 and BRCA2 carrier probabilities for breast and ovarian cancer cases unselected for family history

	**Breast cancer**		**Ovarian cancer**	
**Age at onset (years)**	**BRCA1**	**BRCA2**	**BRCA1**	**BRCA2**
30	0.051 (0.055)	0.050 (0.008)	0.006 (0.001)	0.008 (0.000)
40	0.016 (0.029)	0.020 (0.005)	0.082 (0.026)	0.004 (0.003)
50	0.004 (0.012)	0.015 (0.004)	0.018 (0.039)	0.016 (0.006)
60	0.002 (0.003)	0.012 (0.003)	0.013 (0.013)	0.008 (0.005)
70	0.001 (0.000)	0.008 (0.001)	0.001 (0.004)	0.001 (0.004)

Corresponding probabilities using BRCAPRO within parentheses.

shows the predicted carrier probabilities for a female, who has developed breast or ovarian cancer unselected for FH, together with the corresponding probabilities given by BRCAPRO (CancerGene v3.1, http://www3.utsouthwestern.edu /cancergene). Note that because the relative risk parameters for BRCA1 and BRCA2 were estimated by decade, and the population incidence rates change at every 5-year interval, the mutation prevalence predicted by the BOADICEA model is essentially constant over each 5-year interval. The model predicts that among women who develop breast cancer at age 30 years, approximately 10% carry a BRCA1 or a BRCA2 mutation. The carrier probability drops with age to approximately 1% for women diagnosed at age 70 years. The carrier probabilities given by BRCAPRO are 6.3 and 0.1% for women diagnosed at ages 30 and 70 years, respectively. The same table shows the predicted contributions of BRCA1 and BRCA2 to cases diagnosed with ovarian cancer. The contribution of BRCA1 is low below age 40 years, highest at age 40 years and declines thereafter. The contribution of BRCA2 mutations to ovarian cancer is predicted to be highest at age 50 years (1.6%). This later peak for BRCA2 is consistent with observations from previous studies (Risch *et al*, 2001). The BRCAPRO predicted carrier probabilities demonstrate similar patterns.

The carrier probabilities for an individual who has developed breast cancer and has a mother diagnosed with breast cancer at different ages are shown in Table 3

**Table 3:** Predicted BRCA1/2 carrier probabilities for a breast cancer case whose mother developed breast cancer

	**Age at onset of breast cancer for mother (years)**				
**Index age at onset (years)**	**30**	**40**	**50**	**60**	**70**
*30*					
BRCA1	0.400 (0.566)	0.223 (0.415)	0.075 (0.244)	0.045 (0.103)	0.042 (0.049)
BRCA2	0.318 (0.036)	0.221 (0.034)	0.196 (0.037)	0.153 (0.030)	0.102 (0.020)
					
*40*					
BRCA1	0.212 (0.407)	0.087 (0.270)	0.025 (0.144)	0.014 (0.056)	0.013 (0.025)
BRCA2	0.211 (0.033)	0.107 (0.028)	0.082 (0.027)	0.062 (0.021)	0.041 (0.013)
					
*50*					
BRCA1	0.056 (0.228)	0.020 (0.137)	0.006 (0.067)	0.003 (0.024)	0.003 (0.011)
BRCA2	0.180 (0.035)	0.080 (0.027)	0.061 (0.024)	0.047 (0.018)	0.032 (0.011)
					
*60*					
BRCA1	0.023 (0.079)	0.008 (0.043)	0.002 (0.020)	0.001 (0.007)	0.001 (0.003)
BRCA2	0.134 (0.028)	0.058 (0.020)	0.045 (0.017)	0.036 (0.012)	0.025 (0.007)
					
*70*					
BRCA1	0.019 (0.022)	0.006 (0.011)	0.002 (0.005)	0.001 (0.001)	0.001 (0.000)
BRCA2	0.080 (0.016)	0.034 (0.011)	0.028 (0.009)	0.024 (0.006)	0.017 (0.004)

Corresponding probabilities using BRCAPRO within parentheses.

. Both BRCA1 and BRCA2 carrier probabilities decrease with increasing age at diagnosis of the index individual and her mother. The decrease is more marked for BRCA1 than for BRCA2, so that the prevalence of BRCA1 mutations is higher than BRCA2 at younger ages but lower at older ages. The BRCA1 probabilities are consistently lower than the BRCAPRO predictions, particularly at young ages, while the BRCA2 probabilities are markedly higher.

Table 4

**Table 4:** Predicted BRCA1/2 carrier probabilities for a breast cancer case whose mother and sister have developed breast cancer

	**Age of BC onset of index: 30years**					**Age of BC onset of index: 40 years**					**Age of BC onset of index: 50 years**				
	**Age of BC onset of sister (years)**					**Age of BC onset of sister (years)**					**Age of BC onset of sister (years)**				
Age of BC onset of mother (years)	**30**	**40**	**50**	**60**	**70**	**30**	**40**	**50**	**60**	**70**	**30**	**40**	**50**	**60**	**70**
*30*															
BRCA1	0.577	0.497	0.275	0.239	0.288	0.467	0.367	0.170	0.127	0.140	0.186	0.126	0.047	0.031	0.033
BRCA2	0.387	0.427	0.575	0.544	0.429	0.403	0.396	0.453	0.393	0.287	0.510	0.428	0.405	0.336	0.243
															
*40*															
BRCA1	0.498	0.389	0.179	0.135	0.149	0.367	0.231	0.084	0.055	0.054	0.126	0.064	0.020	0.012	0.012
BRCA2	0.427	0.419	0.476	0.411	0.300	0.397	0.308	0.281	0.218	0.147	0.426	0.267	0.215	0.165	0.111
															
*50*															
BRCA1	0.274	0.179	0.065	0.044	0.047	0.169	0.084	0.026	0.016	0.015	0.046	0.020	0.006	0.003	0.003
BRCA2	0.577	0.478	0.444	0.364	0.262	0.454	0.281	0.224	0.171	0.115	0.402	0.214	0.165	0.127	0.086
															
*60*															
BRCA1	0.237	0.134	0.044	0.028	0.029	0.127	0.054	0.016	0.009	0.009	0.031	0.012	0.003	0.002	0.002
BRCA2	0.549	0.414	0.365	0.295	0.210	0.394	0.219	0.171	0.132	0.089	0.334	0.165	0.127	0.099	0.068
															
*70*															
BRCA1	0.286	0.148	0.046	0.029	0.028	0.140	0.054	0.015	0.009	0.008	0.033	0.012	0.003	0.002	0.002
BRCA2	0.433	0.302	0.262	0.209	0.146	0.288	0.147	0.115	0.089	0.060	0.241	0.111	0.086	0.068	0.047
	**Age of BC onset of index: 60 years**					**Age of BC onset of index: 70years**									
	**Age of BC onset of sister (years)**					**Age of BC onset of sister (years)**									
**Age of BC onset of mother**	**30**	**40**	**60**	**60**	**70**	**30**	**40**	**50**	**60**	**70**					
*30*															
BRCA1	0.100	0.061	0.021	0.013	0.013	0.101	0.057	0.019	0.011	0.011					
BRCA2	0.439	0.340	0.312	0.260	0.186	0.279	0.207	0.194	0.164	0.117					
															
*40*															
BRCA1	0.061	0.028	0.008	0.005	0.005	0.057	0.024	0.007	0.004	0.004					
BRCA2	0.339	0.193	0.155	0.122	0.083	0.206	0.111	0.091	0.074	0.051					
															
*50*															
BRCA1	0.021	0.008	0.002	0.001	0.001	0.019	0.007	0.002	0.001	0.001					
BRCA2	0.309	0.154	0.120	0.096	0.066	0.192	0.091	0.073	0.060	0.042					
															
*60*															
BRCA1	0.013	0.005	0.001	0.001	0.001	0.011	0.004	0.001	0.001	0.001					
BRCA2	0.256	0.120	0.095	0.077	0.054	0.161	0.073	0.059	0.049	0.035					
															
*70*															
BRCA1	0.013	0.005	0.001	0.001	0.001	0.011	0.004	0.001	0.001	0.001					
BRCA2	0.183	0.082	0.065	0.054	0.038	0.115	0.050	0.041	0.035	0.025					

BC=breast cancer.

shows the predicted BRCA1/2 mutation carrier probabilities for breast cancer cases with an affected mother and sister at different ages. As expected, the carrier probabilities are higher when an individual has two relatives diagnosed with the disease as compared with one affected relative. The BRCA2 carrier probabilities are higher than the BRCA1 probabilities, except where at least two of the cases are diagnosed below age 40 years. The difference is particularly marked when all cases are diagnosed at ages 50 years and over, when the BRCA1 carrier probability is low.

### Predicted FRRs

To investigate the consistency of our model with results from epidemiological studies, we computed the FRRs predicted by our model. Table 5

**Table 5:** Age-specific breast cancer familial relative risks associated with family history in a first-degree relative (mother)

	**Predicted relative risk**			
**Age (years)**	**BOADICEA**	**Claus *et al***	**BRCAPRO**	**Observed^a^**
25	3.36	10.33	2.87	
30	5.66	6.13	2.24	5.7
35	2.47	5.63	1.76	
40	2.18	2.32	1.33	2.0
45	1.92	2.00	1.16	
50	1.87	1.51	1.08	1.6
55	1.80	1.37	1.03	
60	1.76	1.10	1.01	
65	1.70	1.07	1.00	1.4
70	1.65	1.05	1.00	

For the predicted relative risks, we assume the same age diagnosis for the mother and the index case.

a Collaborative Group on Hormonal Factors in Breast Cancer (2001).

shows the FRR of breast cancer at different ages, associated with a mother diagnosed with breast cancer. Also shown for comparison are the FRRs published in the largest combined analysis of familial risks (Collaborative Group in Hormonal Factors in Breast Cancer, 2001), and the corresponding predictions using the Claus *et al*, (1991) model and BRCAPRO. The Claus model was implemented in MENDEL using the parameter estimates reported in Claus *et al* (1991). The FRR predicted by our model decreases with age from 5.66 at age 30 years to 1.65 at age 70 years. The predicted relative risk at age 25 years (3.36) is lower than that at age 30 years, but the incidence rates in carriers are particularly imprecise at this age (Antoniou *et al*, 2002). The predicted relative risk in the 40–49 years age group is very similar to the published estimates from the Collaborative Group analysis. At older ages the predictions are slightly higher than the observed values. The Claus model predicts a higher relative risk than BOADICEA at ages 45 years and below, but a lower relative risk at ages 50 years and above. There is a marked difference between the Claus model predictions and the Collaborative group estimates at ages 60 years and above. The relative risks predicted by BRCAPRO are much lower than the Collaborative group estimates at all ages. They are also lower than the BOADICEA and Claus *et al* predictions. The intraclass correlation coefficient between the BOADICEA predictions and the Collaborative group estimates is estimated to be 0.99. The corresponding coefficients for the Claus *et al* model and BRCAPRO are 0.99 and 0.35.

### Predicted annual incidence in monozygotic twins and sisters of breast cancer patients

Peto and Mack (2000) have noted that the risk of risk of contralateral breast cancer is approximately constant at 0.7% per annum, regardless of the age at diagnosis of first cancer or the time since the first cancer. They further note that breast cancer in monozygotic twins of breast cancer patients is approximately constant, at 1.4% per annum, following the age at diagnosis of the co-twin, and that the risk of breast cancer to sisters of breast cancer cases is approximately constant at 0.35% per annum. We have evaluated the consistency of BOADICEA model with these observations.

Table 6

**Table 6:** Predicted annual incidence (%), in a sister of a breast cancer patient, at ages older than the index patient's age at diagnosis

	**Age of sister (years)**				
**Index age at diagnosis (years)**	**30–39**	**40–49**	**50–59**	**60–69**	**70–79**
30	0.14	0.30	0.35	0.41	0.44
	0.10(B)	0.24(B)	0.30(B)	0.34(B)	0.33(B)
	0.20(C)	0.43(C)	0.39(C)	0.42(C)	0.80(C)
	(0.33,0.14)	(0.78, 0.25)	(0.49, 0.52)	(0.78)	(—)
					
35		0.25	0.32	0.39	0.42
		0.22(B)	0.29(B)	0.33(B)	0.33(B)
		0.42(C)	0.38(C)	0.41(C)	0.77(C)
		(0.40,0.34)	(0.43,0.39)	(0.24, 0.44)	(0.69, 0.20)
					
45			0.30	0.38	0.41
			0.27(B)	0.32(B)	0.33(B)
			0.25(C)	0.31(C)	0.54(C)
			(0.28,0.40)	(0.34, 0.23)	(0.42, 0.45)
					
55				0.37	0.41
				0.32(B)	0.32(B)
				0.28(C)	0.46(C)
				(0.36)	(0.48)

B=BRCAPRO predictions; C=Claus *et al* predictions.

Within parentheses are the reported annual incidence rates in the two studies of the analysis of Peto and Mack (2000).

shows the predicted incidence in a sister of a breast cancer patient, at ages older than the age at onset of the patient's breast cancer. Under the BOADICEA model, most of the incidence rates are in the range 0.3–0.45%, in line with the suggestion of Peto and Mack. However, the incidence rates increase slightly with age. The most marked discrepancy is below age 40 years, for cases diagnosed at age 30 years, where the predicted rate is 0.16%. For comparison the table includes the reported incidence rates in mothers and sisters in the two studies reported by Peto and Mack (2000). Of the 10 cells where there are estimates from both studies, the predicted incidence lies between the two estimates in five, lower than both estimates in four and higher than both in one. The intraclass correlation coefficient between the BOADICEA predictions and the Peto and Mack estimates is 0.36. Table 6 also shows the predicted annual incidence under the Claus *et al* model and BRCAPRO. The intraclass correlation coefficients for these predictions with the Peto and Mack estimates were 0.34 and 0.07, respectively, indicating that the agreement between the BRCAPRO predictions and the observed values is poorer than the corresponding BOADICEA or Claus *et al* predictions.

The predicted incidence in the monozygotic twin following the diagnosis of breast cancer in their co-twin is shown in Table 7

**Table 7:** Predicted annual breast cancer incidence (%) in a monozygotic twin in the years after the diagnosis in the first twin

	**Years since first twin's diagnosis (years)**			
**First twin age (years)**	**<5**	**5–9**	**10–14**	**15+**
35	0.25	0.43	0.55	0.68
	0.17(B)	0.26(B)	0.30(B)	0.34(B)
	0.34(C)	0.77(C)	0.70(C)	0.85(C)
				
40	0.40	0.52	0.55	0.70
	0.22(B)	0.26(B)	0.29(B)	0.33(B)
	0.46(C)	0.42(C)	0.41(C)	0.56(C)
				
45	0.49	0.53	0.58	0.70
	0.24(B)	0.27(B)	0.30(B)	0.33(B)
	0.38(C)	0.37(C)	0.34(C)	0.57(C)
				
50	0.52	0.57	0.65	0.71
	0.26(B)	0.29(B)	0.32(B)	0.33(B)
	0.31(C)	0.29(C)	0.36(C)	0.52(C)
				
55	0.55	0.63	0.68	0.71
	0.28(B)	0.31(B)	0.33(B)	0.33(B)
	0.26(C)	0.34(C)	0.32(C)	0.57(C)

B=BRCAPRO predictions; C=Claus *et al* predictions.

. The predicted incidence under the BOADICEA model is approximately twice that of the corresponding incidence in a sister. Moreover, as in the case of sisters, the incidence is not constant over the years after the first twin's diagnosis, but increases slightly over time. Similar patterns are observed for the predicted incidence under the Claus *et al* model. However, under BRCAPRO the predicted incidence in a monozygotic twin of a breast cancer patient is very similar to the incidence in sisters (Table 6).

Table 8

**Table 8:** Predicted annual incidence (%) of contralateral breast cancer

	**Years since first breast cancer (years)**		
**Age at first cancer (years)**	**<5**	**5–9**	**10+**
35	0.15 (0.18)	0.25 (0.42)	0.37 (0.64)
40	0.23 (0.26)	0.30 (0.25)	0.37 (0.42)
45	0.29 (0.24)	0.30 (0.24)	0.37 (0.43)
50	0.31 (0.20)	0.32 (0.19)	0.39 (0.40)
55	0.34 (0.18)	0.36 (0.22)	0.39 (0.43)

Within parentheses are the Claus *et al* predictions.

shows the annual incidence of contralateral breast cancer predicted by the BOADICEA model, as modified to allow for second cancers. Overall the contralateral incidence rate is about half the incidence in a monozygotic twin of a breast cancer case in the years after the first twin's diagnosis. Again, the incidence increases slightly with the years since diagnosis of the first cancer, although the increase is only marked for cases diagnosed below age 45 years. The same table shows the predictions under the Claus *et al* model, which was modified in a similar manner to allow for contralateral breast cancer. Similar patterns were observed to the BOADICEA predictions. The predicted incidence of contralateral breast cancer under the Claus model is in general somewhat lower than the BOADICEA incidence in the 10 years just after the first diagnosis, but higher thereafter.

### Genetic modification of risk in BRCA1 and BRCA2 carriers

**Figure 1:**
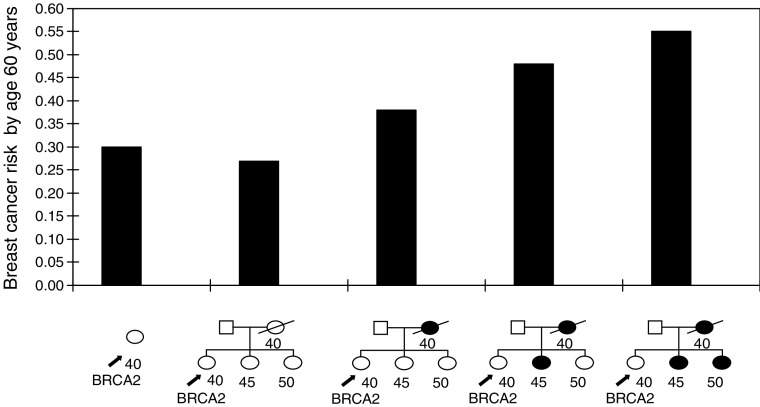
Predicted risks of breast cancer by age 60 years, to a 40-year-old female BRCA2 mutation carrier according to her FH of breast cancer.

Under the BOADICEA model, the polygenic component is assumed to act on both carriers and noncarriers of BRCA1 and BRCA2 mutations. In the analyses of Antoniou *et al* (2002), the variance of the polygenic component did not vary significantly between carriers and noncarriers. A consequence of this model is that the absolute risk of breast cancer in a carrier will depend on FH. To illustrate this, we calculated the risks to a healthy 40-year-old female BRCA2 mutation carrier according to her FH of breast cancer, as illustrated in Figure 1

. For a BRCA2 carrier without any information on FH, the predicted absolute risk of breast cancer by age 60 years is 30%. If we assume that the woman has two sisters unaffected at ages 45 and 50 years, respectively, and that her mother died at age 40 years without developing breast cancer, then her predicted risk reduces to 27%. If we assume that her mother had developed breast cancer at age 40 years, then the predicted risk increases to 38%. If in addition to the mother, one sister is assumed to have developed breast cancer at age 45 years, the predicted breast cancer risk by age 60 years rises to 48%. Finally, if both of her sisters and her mother are assumed to have developed breast cancer, her risk rises to 55%.

### Predictions in high-risk families

**Figure 2:**
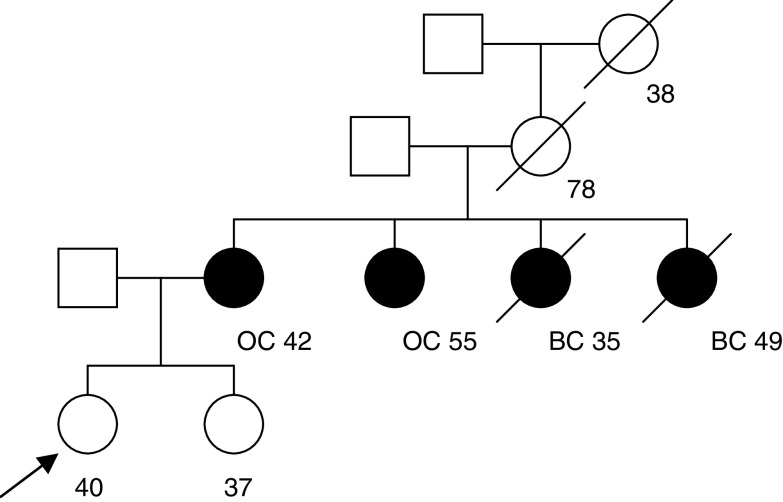
Family 1 with information on age at last follow-up and age at diagnosis. Index indicated by arrow. BC=breast cancer; OC=ovarian cancer.

**Figure 3:**
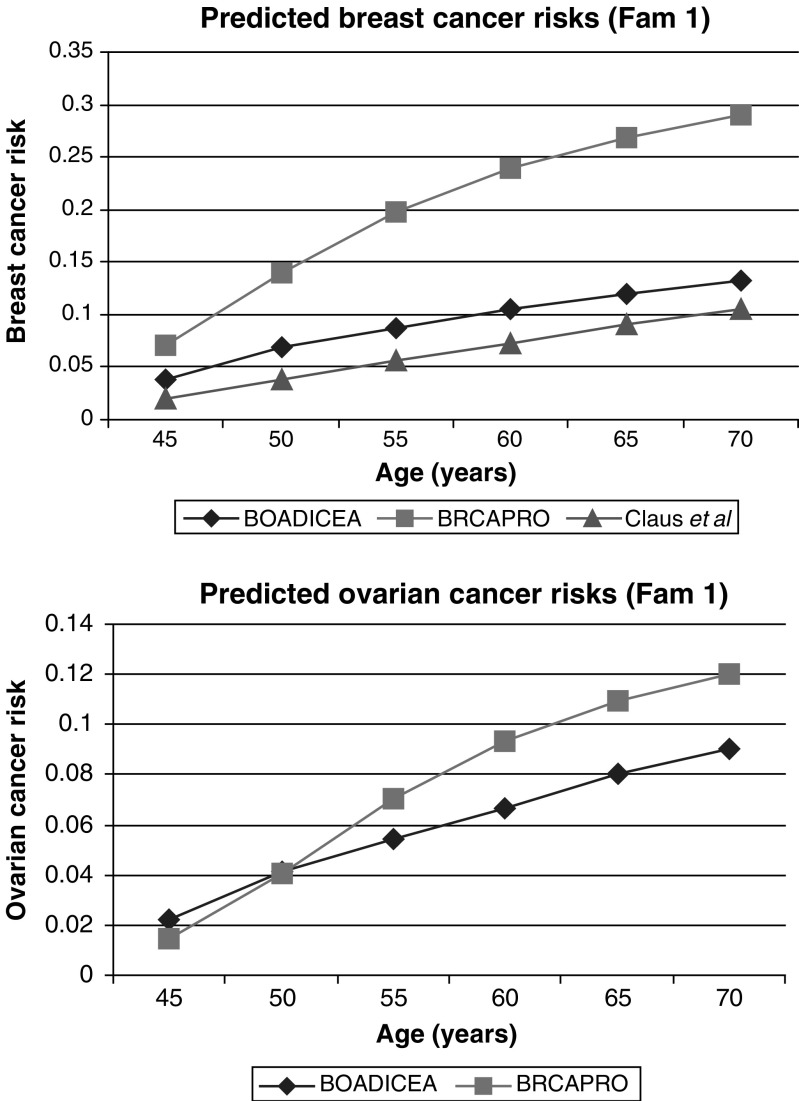
Predicted cancer risks for the index female of family 1 under the following models: BOADICEA, BRCAPRO (CancerGene v3.1, http://www3.utsouthwestern.edu /cancergene) and Claus *et al* (1991).

To examine the predictions of our model in women with a strong FH, we have considered two families where the index case was participating in the MARIBS study of MRI screening for women at a high risk of breast cancer (Brown *et al*, 2000; Leach *et al*, 2002). These families are thus examples of ‘high-risk’ families seen at cancer genetics clinics. For these families, we have compared our predictions with those given by BRCAPRO (CancerGene v3.1, http://www3.utsouthwestern.edu /cancergene) and the Claus model (Claus *et al*, 1991). The Claus model takes into account breast cancer but not ovarian cancer occurrence in the family. In the first example, the consultant is 40 years old and unaffected with cancer. Her mother and one maternal aunt developed ovarian cancer and another two maternal aunts developed breast cancer at ages 35 and 49 years (Figure 2

). The BOADICEA model predicts that she carries a BRCA1 mutation with a probability of 40.9% and a BRCA2 mutation with a probability of 1.3%. In this example, the carrier probabilities given by BRCAPRO are similar: 39.3% and 1.6% for BRCA1 and BRCA2, respectively. The predicted risk of breast cancer by age 70 years under the BOADICEA model is 13% (Figure 3

). This is similar to that predicted by the Claus model (11%) but much lower than that predicted by BRCAPRO (29%). BOADICEA also predicts a lower risk of ovarian cancer (9% by age 70 years) than BRCAPRO (12%).

**Figure 4:**
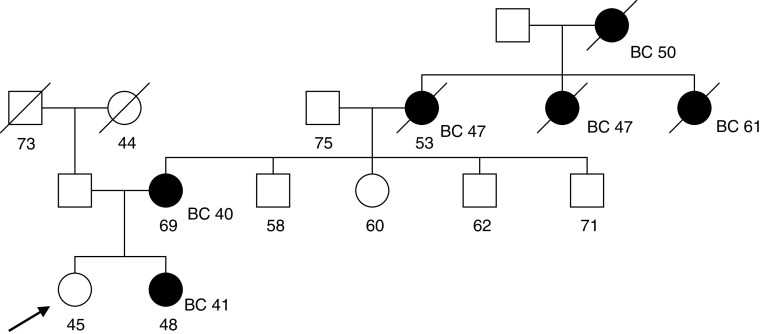
Family 2 with information on age at last follow-up and age at diagnosis. Index indicated by arrow. BC=breast cancer.

The second family is shown in Figure 4

**Figure 5:**
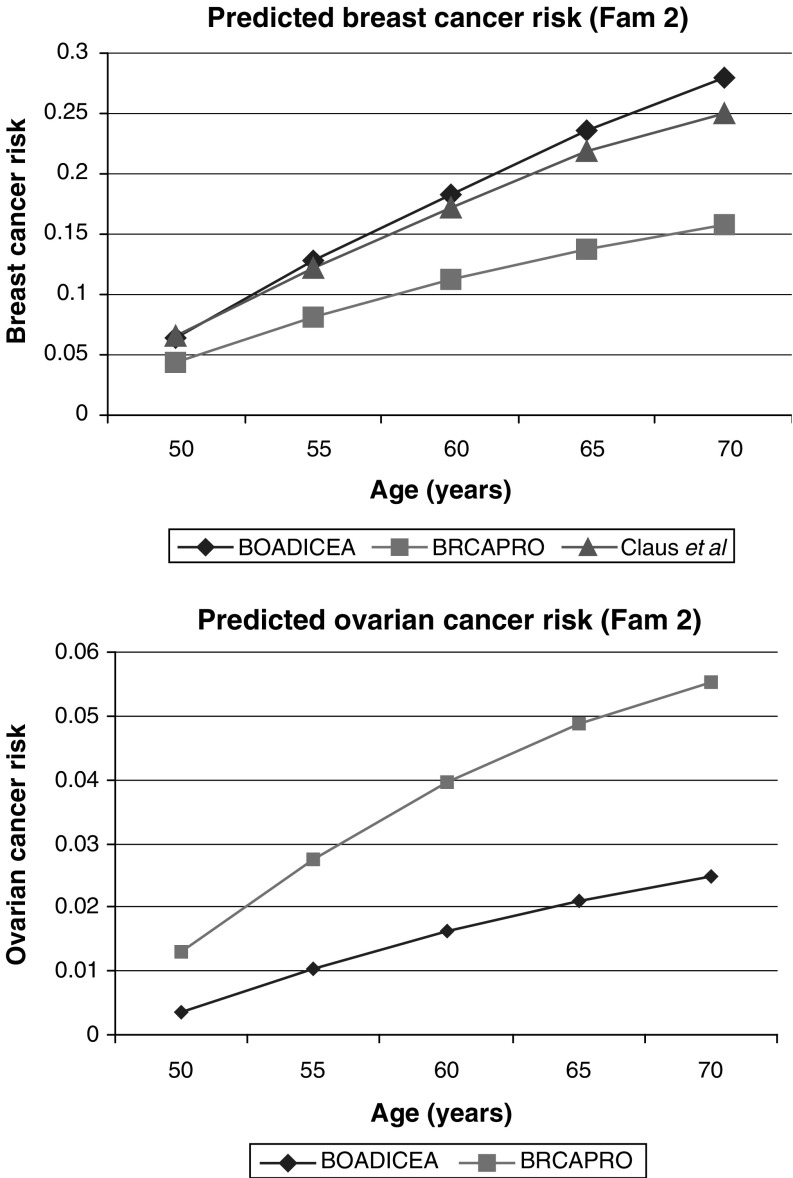
Predicted cancer risks for the index female of family 2 under the following models: BOADICEA, BRCAPRO (CancerGene v3.1, http://www3.utsouthwestern.edu /cancergene) and Claus *et al* (1991).

. The female seeking genetic counselling is 45 years old and has several relatives diagnosed with breast cancer, including her mother, sister, maternal grandmother and two maternal great aunts. Our model predicts that she carries a BRCA1 mutation with probability of 2.3% and a BRCA2 mutation with probability 30.1%. These compare with BRCAPRO probabilities of 17% for BRCA1 and 2.4% for BRCA2. The cumulative age-specific cancer risks for this individual are summarised in Figure 5

. BOADICEA gives the highest breast cancer risk by age 70 years (28%) by age 70 and BRCAPRO the lowest (16%). The age-specific breast cancer risks given by the Claus *et al* model (25% by age 70 years) are similar to those predicted under our model.

**Figure 6:**
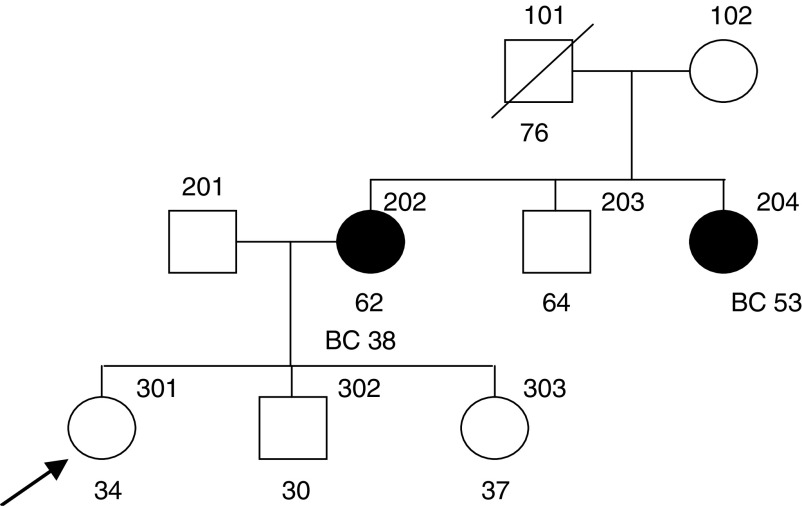
Family 3 with information on age at last follow-up and age at diagnosis. Index indicated by arrow. BC=breast cancer.

Although for the age-specific cancer risks given by the Claus *et al* model were similar to those given by BOADICEA in both the above examples, this is not true generally. For example, consider individual 301 in Figure 6

, who is unaffected at age 34 years. Under BOADICEA, her estimated risk by age 70 years is approximately 12%, whereas the predicted risk under the Claus model is 24%.

## DISCUSSION

The BOADICEA model is a general model of breast cancer susceptibility that can provide estimates of cancer risks and carrier probabilities to women with a FH of breast/ovarian cancer. However, before using such a model in clinical practice, it is important to evaluate the accuracy of its predictions in independent data sets that were not used to derive the model. A number of such validation studies have recently been reported for other models, including BRCAPRO and Claus (Amir *et al*, 2003, Marroni *et al*, 2004, Evans *et al*, 2004), and have identified some model deficiencies while suggesting ways of refining the algorithms. In this paper, we have examined the frequencies of the BRCA1 and BRCA2 mutations in unselected series of breast cancer patients, and the FRRs of breast cancer, predicted by the BOADICEA model, since these can be compared with empirical observations.

Several studies have investigated the prevalence of BRCA1 and BRCA2 mutation in series of breast cancer cases unselected for FH in nonfounder populations (i.e. excluding populations such as the Ashkenazi Jewish or Icelandic populations where there are prevalent founder mutations) (Langston *et al*, 1996; Peto *et al*, 1999; Hopper *et al*, 1999; Malone *et al*, 2000). The pattern of predicted age-specific contributions of BRCA1 and BRCA2 mutations to breast cancer (Table 2) is generally in line with the results of the population studies, where the contribution is highest at young ages at onset and thereafter it decreases. Direct comparison with empirical observations is complicated by the fact that these studies have used different screening techniques, with different sensitivities. However, in the only other UK study to screen the coding sequences of both genes, Peto *et al* (1999) used the same technique (CSGE) as the studies on which BOADICEA was based. They estimated that 3.5% of women diagnosed under age 36 years carried a BRCA1 mutation and 2.4% carried a BRCA2 mutation. Assuming similar mutation detection sensitivity (∼70%) for CSGE used in the development of BOADICEA, the corresponding contributions predicted by our model are 3.4% for BRCA1 and 1.3% for BRCA2, very similar for BRCA1 but somewhat lower for BRCA2 than the observed proportions. In the Peto *et al* study (Peto *et al*, 1999), BRCA1 and BRCA2 were estimated to account for 1.9% and 2.2% of unselected breast cancers diagnosed between ages 36 and 45 years. These are somewhat higher than the contributions predicted by BOADICEA (1.3% for BRCA1, 1.5% for BRCA2) but consistent given the small number of mutations. By comparison, the predictions under the BRCAPRO model are 3.7% and 0.6% for BRCA1 and BRCA2, respectively, for cases diagnosed under age 36 years and 1.8% and 0.4% for breast cancers diagnosed between ages 36 and 45 years. Thus, the BRCA2 predictions from BOADICEA are much closer to the observed values. The prevalence of mutations in breast and ovarian cancer cases in the Ashkenazi and Icelandic populations is higher; to be applicable to these populations, the model will need to be adapted to use higher allele frequencies. Alterations to the allele frequencies may also be required for other populations with more limited numbers of mutations, for example, the Polish, French Canadian and Dutch populations.

We also compared the age-specific breast cancer risks to women with a first-degree FH of breast cancer with those reported in epidemiological studies. The age-specific FRRs predicted by our model are similar to the estimates of the meta-analysis reported by the Collaborative Group in Hormonal Factors in Breast Cancer (2001), with a gradual decline in relative risk with age of the consultand and age at diagnosis of the relative. The main difference is at older ages, where the predicted relative risks are somewhat greater than the observed values. By comparison, the Claus *et al* (1991) model fits poorly at older ages, particularly beyond age 60 years, while the predicted relative risks from BRCAPRO are substantially lower than the observed values at all ages. The latter result was expected as BRCAPRO takes into account only BRCA1 and BRCA2, which explain about 20% of the excess FRR of breast cancer (Parmigiani *et al*, 1998; Easton, 1999; Peto *et al*, 1999).

Peto and Mack (2000) examined the risks of contralateral breast cancer and noted that the incidence rates were approximately 0.7% per annum, independent of age. They also noted that the risks of breast cancer in monozygotic twins and sisters of cases were also approximately constant over time after the age at diagnosis of their relative, at approximately 1.4 and 0.35%, respectively. They postulated that such effects might be due to a model in which the risk of breast cancer reaches a constant high level at a genetically determined age. This concept is different from that underlying the polygenic model in BOADICEA, in which multiple genes ‘interact’ multiplicatively to increase risk at all ages. It was therefore particularly interesting to examine the predictions of these risks made by the BOADICEA model. The predicted annual incidence of breast cancer in sisters of breast cancer patients at ages older than the index patient's age at diagnosis is quite consistent with the studies reported by Peto and Mack (2000), although the incidence does increase slightly with age. The predicted risks to MZ twins of breast cancer cases were, however, less than those reported by Peto and Mack (2000). Unfortunately, the data on age-specific risks to MZ twins are limited so it is unclear if this discrepancy is real.

The predicted incidence of contralateral breast cancer is also markedly lower than that summarised by Peto and Mack (2000), based on the Connecticut Tumor Registry. Our estimates are in fact closer to those reported by Vaittinen and Hemminki (2000), based on data from the Swedish Family Cancer Registry. They estimated that the contralateral breast cancer incidence is around 0.8% at age 30 years and decreases to a constant level of around 0.4% at ages 50 years and above. Contralateral breast cancers were not a component of the original BOADICEA model but have been incorporated into the model by assuming that, conditional on genotype, the incidence of a breast cancer in the opposite breast is half the overall incidence of breast cancer at the same age. The underestimation of the contralateral risk by the BOADICEA model, if substantiated, may indicate that additional intraindividual factors influence risk. If so, separate parameters to allow for the higher rate of second cancers may be required.

The current model was derived using families ascertained through breast cancer probands, and included both mutation-positive and mutation-negative cases (Antoniou *et al*, 2001, 2002). However, the estimated incidence rates in BRCA1 and BRCA2 carriers are based on a relatively limited number of mutation-positive families (62 in total), so these incidence rates are somewhat imprecise. More precise penetrance estimates have been derived in a meta-analysis of the families of BRCA1/2 carriers identified through population-based studies of breast and ovarian cancer (Antoniou *et al*, 2003). The BRCA2 penetrance function in our model is very similar to that estimated by Antoniou *et al* (2003), but the BRCA1 risks in our model are lower than the meta-analysis estimates (35 *vs* 65% by age 70 years). These estimates are not directly comparable since the BOADICEA model allows for the polygenic component that modifies the BRCA1 and BRCA2 risks, whereas the estimates of Antoniou *et al* (2003) do not allow for a polygenic component. Moreover, those estimates were based on the risks in relatives of breast cancer patients identified through population-based studies, while the BOADICEA risks are the average risks for the whole population. Nevertheless, difference in the BRCA1 risks estimated by the BOADICEA model and the meta-analysis of Antoniou *et al* (2003) suggests that the BOADICEA model may have underestimated the BRCA1 risk. Refinement of the BOADICEA model by refitting using additional family data is in progress.

We have demonstrated that the predicted cancer and carrier risks in some families differ markedly from those predicted under other models. In our comparisons, we concentrated on the two widely used genetic models of Claus *et al* (1991) and Parmigiani *et al* (1998) (BRCAPRO). Other models are based on empirical summaries of FH rather than pedigree analysis of a genetic model (Gail *et al*, 1989; Shattuck-Eidens *et al*, 1995; Couch *et al*, 1997; Apicella *et al*, 2003). We did not consider these models here since they are not directly comparable; however, from our experience these models can also give predictions that differ substantially from BOADICEA (results not shown).

The model of Claus *et al* (1991) assumes a single dominant gene with an allele frequency of 0.33%. This is clearly a considerable oversimplification since there is no allowance for the differences in risks between BRCA1 and BRCA2 carriers (Ford *et al*, 1998; Malone *et al*, 2000; Antoniou *et al*, 2003) or for other lower risk genes. In the examples we examined, the breast cancer risks predicted by the Claus *et al* (1991) model were sometimes similar to those predicted by BOADICEA (as in Figures 3 and 5) but sometimes quite different (as for family 3 in Figure 6).

The BRCAPRO model is closer to BOADICEA in that BRCA1 and BRCA2 are modelled separately. The differences between BOADICEA and BRCAPRO can be explained partly by the different penetrance functions and allele frequencies for BRCA1 and BRCA2 assumed by the two models. Specifically, BRCAPRO assumes BRCA1 mutations to be more prevalent than BRCA2 (Berry *et al*, 2002), whereas BRCA1 and BRCA2 mutations have similar population frequencies in BOADICEA, with BRCA2 being slightly more prevalent. We investigated the effect on predictions by changing the allele frequencies in BRCAPRO to those assumed by the BOADICEA model (Antoniou *et al*, 2002). This resulted in somewhat higher predicted prevalence for BRCA2 mutations among unselected breast cancer patients. BRCA2 mutations were predicted to account for 2.4% of the patients diagnosed at age 30 years (compared to 0.8% previously) and for 0.4% of the patients diagnosed at age 70 years (compared to 0.1% previously). Moreover, for ages at diagnosis of 50 years and over BRCA2 mutations were predicted to be more prevalent than BRCA1 mutations, a feature similar to BOADICEA. Changing the allele frequencies in BRCAPRO did not have a marked effect on the predicted FRRs, which were very similar to the predictions in Table 5. Furthermore, the extent of FH that the models consider may also be important in explaining the differences between BOADICEA and BRCAPRO. BRCAPRO does not consider FH information on relatives more distant than second degree, whereas the present model can incorporate all available relatives. In practice however, including data on third- (or higher) degree relatives may bias the predictions as self-reporting FH can be less reliable on these relatives (Thompson and Schildkraut, 1991). As for the Claus *et al* model, however, the most important difference is that BRCAPRO makes no allowance for other susceptibility genes other than BRCA1 and BRCA2.

Several improvements can be made to the present model. An obvious deficiency (also present in other models) is that the genotype-specific incidence rates are computed for broad age categories (mostly 5 years). Thus, for example, the incidence rates are assumed constant over the period 30–34, but different from those at 29 years or age 35 years. This leads to substantial steps in carrier probabilities and predicted risks that could be improved by smoothing. As more data become available, we plan to refit the model in order to obtain more accurate estimates of the BRCA1 and BRCA2 allele frequencies and penetrances and of the polygenic component. In addition to the breast and ovarian cancer risks, there is evidence that BRCA1 and BRCA2 mutations confer increased risks of other cancers such as prostate cancer and pancreatic cancer (The Breast Cancer Linkage Consortium, 1999; Thompson and Easton, 2002). The model can be extended straightforwardly to incorporate such information. BRCA1- and BRCA2-associated breast cancer tumours have also been reported to have different pathological characteristics from one another, and also from sporadic and other familial tumours (Breast Cancer Linkage Consortium, 1997; Lakhani *et al*, 1998; Lakhani *et al*, 2000; Lakhani *et al*, 2002). Incorporating pathological information into the model would improve the accuracy of carrier prediction. In principle, the model can also be extended to incorporate nongenetic risk factors, such as parity, breast feeding and age at menopause. This, however, requires precise estimates of these effects in BRCA1 and BRCA2 carriers.

Finally, the model can be extended to account for the effects of other susceptibility genes. The polygenic component in BOADICEA represents the combined effects of low-penetrance breast cancer susceptibility genes. To date, only two low-penetrance breast cancer susceptibility genes, ATM and CHEK2, have been reliably identified (Swift *et al*, 1990; The CHEK2-Breast Cancer Consortium 2002). In the case of CHEK2, the truncating variant 1100delC confers a relative risk of breast cancer approximately two-fold; this relative risk appears to be independent of FH, and this gene fits well as a component of a multiplicative polygenic model. As other such genes are identified, the model can be extended to allow explicitly for their effects.
